# Phthalates exposure and gastrointestinal health: a comprehensive review of toxic mechanisms and emerging mitigation strategies

**DOI:** 10.1016/j.crtox.2026.100322

**Published:** 2026-10-24

**Authors:** Choirul Anwar, Pitchurajan Krishna Perumal, Ping-Chi Hsu, Ching-Shu Lai

**Affiliations:** a Department of Seafood Science, National Kaohsiung University of Science and Technology, Kaohsiung, 81157, Taiwan; b Department of Safety, Health and Environmental Engineering, National Kaohsiung University of Science and Technology, Kaohsiung, 81157, Taiwan

**Keywords:** Phthalates, Environmental toxicants, Gastrointestinal toxicity, Emerging organic pollutants, Barrier dysfunction

## Abstract

Phthalates are widely used plasticizers that can leach into food products, making dietary exposure a significant food safety concern. Increasing evidence indicates that phthalates adversely affect gastrointestinal health and contribute to the development of inflammatory bowel disease (IBD), metabolic disorders, diabetes, and cardiovascular disease. Mechanistic studies demonstrate that phthalates disrupt gut microbiota composition, diversity, and metabolic activity, compromising microbial barrier function integrity and facilitating pathogen translocation. They also impair intestinal barrier function by reducing mucus secretion, altering tight junction proteins, and increasing epithelial permeability. Furthermore, phthalates promote intestinal inflammation by enhancing pro-inflammatory cytokine production, suppressing anti-inflammatory responses, and disrupting immune cell homeostasis. Emerging findings reveal detrimental effects on enteroendocrine and enteric glial cells, resulting in altered hormone secretion, impaired gut-brain signaling, and disrupted intestinal motility. This review summarizes current evidence on molecular mechanisms underlying phthalate-induced gastrointestinal toxicity and highlights potential mitigation strategies, including polyphenols, flavonoids, probiotics, prebiotics, and pharmacological interventions.

## Highlights

• Phthalates impair gut microbial, chemical, physical, and immune barriers.
• Phthalate exposure alters gut microbiota and reduces SCFA production.
• Phthalates disrupt tight junction proteins and intestinal morphology.
• Toxicity triggers systemic inflammation and endocrine-glial dysfunction.
• Natural compounds and probiotics serve as effective mitigation strategies.

## Graphical abstract

**Figure:**
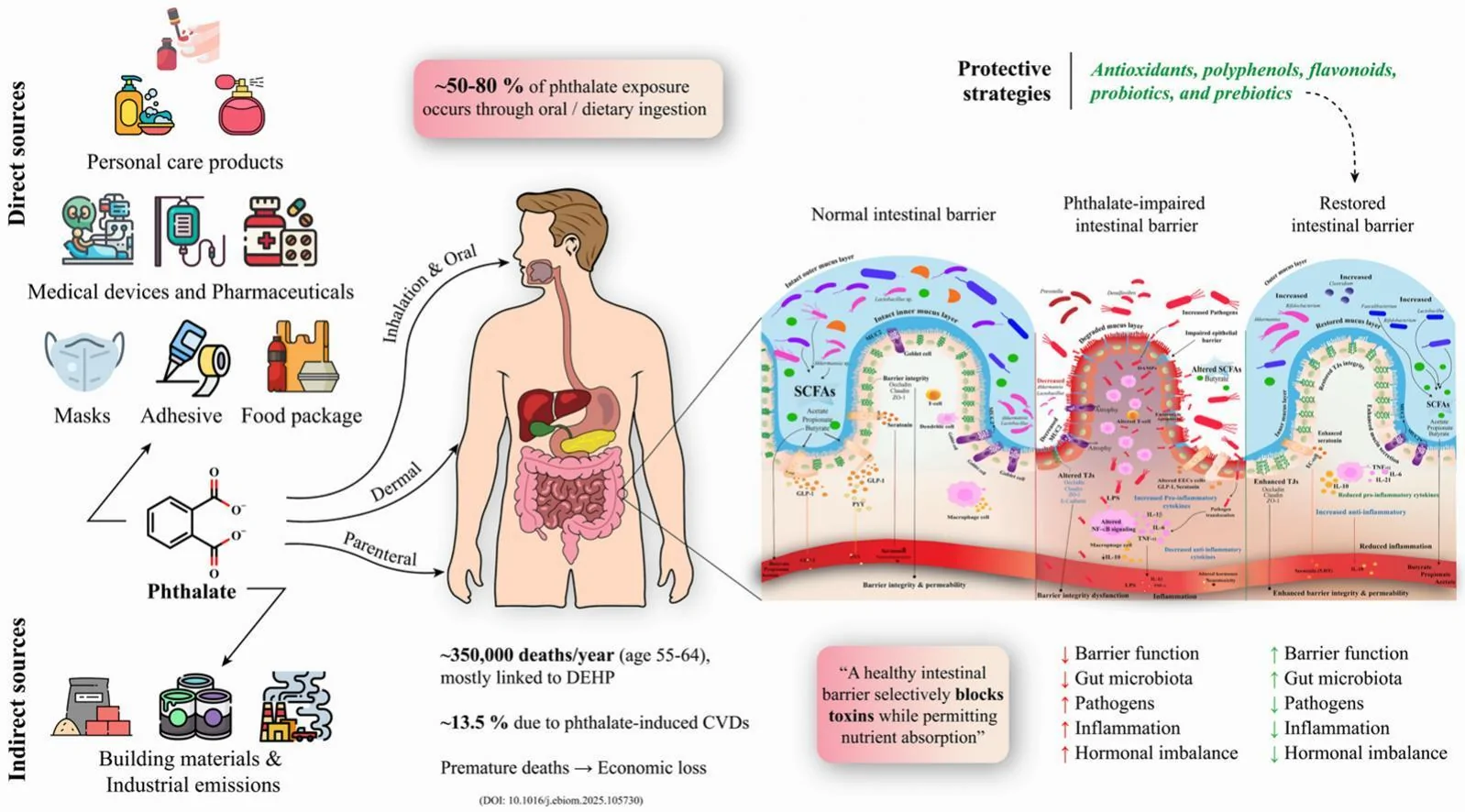

## 1 Introduction

In the modern era, plasticizers are unavoidable, non-volatile compounds that are essential for the production of plastics and elastomers, as they enhance processability, flexibility, and elasticity (Moeini et al., 2021). However, due to weak bonding with polymer matrices and environmental degradation, plasticizers can leach into the environment, resulting in their accumulation in the food chain and subsequent multi-organ damage in humans (Sree et al., 2023). Phthalates are among the most commonly used plasticizers, owing to their favorable physicochemical properties and durability. They are extensively incorporated into consumer products such as food packaging materials, medical devices, cosmetics, and construction materials (Merida et al., 2023; Sree et al., 2023). Globally, approximately 6–8 million tons of phthalates are produced annually for diverse industrial applications, particularly in plastic manufacturing (Sahoo and Kumar, 2023). Driven by increasing demand, global plastic production is projected to reach nearly 500 million tons by 2050 (Crepin et al., 2023). Despite their substantial commercial importance, phthalates are identified as emerging organic pollutants that cause adverse effects on human health, including endocrine disruption, reproductive toxicity, hepatotoxicity, nephrotoxicity, and gastrointestinal toxicity (Hasan et al., 2025).

Phthalates can leach into food products through packaging material, manufacturing process, adulteration, and contaminated water sources, leading to various life-threatening effects and diseases in humans and animals. Although phthalates are semi-volatile compounds, the primary route of human exposure is ingestion, mainly through contaminated food and drinking water. Consequently, the detrimental effects of phthalates on the gastrointestinal (GI) tract are inevitable (Hasan et al., 2025; Zhang and Hou, 2025). Globally, phthalate-induced toxicity is an escalating public health concern that affects individuals across all age groups.

In 2011, the Taiwan Food and Drug Administration reported that two food manufacturing industries illegally used phthalates, particularly Di-(2-ethylhexyl) phthalate (DEHP) and Diisononyl phthalate (DiNP) as clouding agents in packaged foods instead of palm oil to reduce production costs. This incident resulted in endocrine disruption and maternal complications in exposed women (Yang et al., 2013). Furthermore, exposure was associated with impaired intellectual development in school-aged children, specifically affecting linguistic learning and expression abilities (Huang et al., 2017). Collectively, these findings highlight the severity of phthalate-induced health damage irrespective of age or gender and confirm ingestion as a major exposure route, potentially causing harm to internal organs, particularly the GI tract and liver.

The gastrointestinal tract is a highly complex organ system responsible for food digestion, nutrient absorption, and waste elimination (Almamoun et al., 2023). Beyond its digestive functions, the gut plays a critical role in maintaining barrier integrity against pathogens and toxins, regulating immune responses, and preserving endocrine homeostasis (Yang et al., 2022a). The increasing consumption of phthalate-contaminated foods has therefore raised serious concerns regarding gut-related health outcomes. For example, a recent study reported that takeaway foods are highly prone to phthalate contamination, resulting in oxidative stress and DNA damage among college students (Zhong et al., 2022). Additionally, growing evidence suggests that phthalate exposure disrupts gut microbiota composition, leading to microbial dysbiosis and subsequent immune system impairment (Xiong et al., 2020; Zeng et al., 2024). Other studies have linked ingested phthalate exposure to host pathological changes, including reproductive, hepatic, cardiac, and neurodevelopmental disorders (Hasan et al., 2025; Zhang and Hou, 2025). These findings underscore the urgent need to explore effective mitigation strategies to control and prevent phthalate-induced gastrointestinal toxicity.

Accordingly, the present review elucidates the effects of phthalate exposure on gut microbiota functionality and host physiological responses. While the endocrine-disrupting and reproductive toxicities of phthalates have been extensively investigated, comparatively limited attention has been paid to the mechanisms underlying phthalate-induced gastrointestinal toxicity and its impact on gut functions, such as barrier integrity. Emerging research further indicates that phthalate-induced alterations in the gut can trigger metabolic dysfunction and associated metabolic disorders. This review therefore provides critical insights into the pivotal role of the gastrointestinal tract in initiating or exacerbating metabolic disturbances in response to phthalate exposure. Moreover, this review aligns with the United Nations Sustainable Development Goals (SDGs), particularly **SDG 3: Good health and well-being**, by addressing the burden of phthalate-induced GI toxicity and promoting preventive & treatment strategies, and **SDG 12: Responsible consumption and production**, by highlighting human health risks from phthalate-contaminated foods and consumer products, thereby emphasizing safer production, regulation, and chemical management.

## 2 Phthalate classification

Phthalates are classified into two groups based on their molecular weight: high molecular weight phthalates (HMwPs) and low molecular weight phthalates (LMwPs) (Merida et al., 2023; Sree et al., 2023). HMwP consist of long ester chains (typically above 7 carbon atoms), and are widely used as plasticizers in the production of plastics, including polyvinyl chloride (PVC), medical devices, medical implants, and food-packing materials, owing to their flexibility and durability (Saeidnia, 2014; Smith et al., 2022). Common HMwPs include Di-(2-ethylhexyl) phthalate (DEHP), Dicyclohexyl phthalate (DCHP), Diisodecyl phthalate (DiDP), and Diisononyl phthalate (DiNP). In contrast, LMwPs are composed of short ester chains (typically 1–4 carbon atoms) and are extensively used as solvents, fragrance carriers, and plasticizers in various cosmetic and personal care products, such as shampoos, lotions, perfumes, nail polish, and body washes (Su et al., 2019; Merida et al., 2023). Representative LMwPs include dimethyl phthalate (DMP), dibutyl phthalate (DBP), diethyl phthalate (DEP), and benzylbutyl phthalate (BBP). The chemical structures of these phthalates are illustrated in Fig. 1**.** As shown, the structural configurations and functional groups differ among phthalates, resulting in variations in solubility and physicochemical behavior. LMwP are relatively more volatile; however, their volatility decreases in aqueous environments. In contrast, HMwP exhibits a predominantly hydrophobic nature and readily penetrates organic matter, thereby contributing to its toxicity (Sree et al., 2023).

**Fig. 1:**
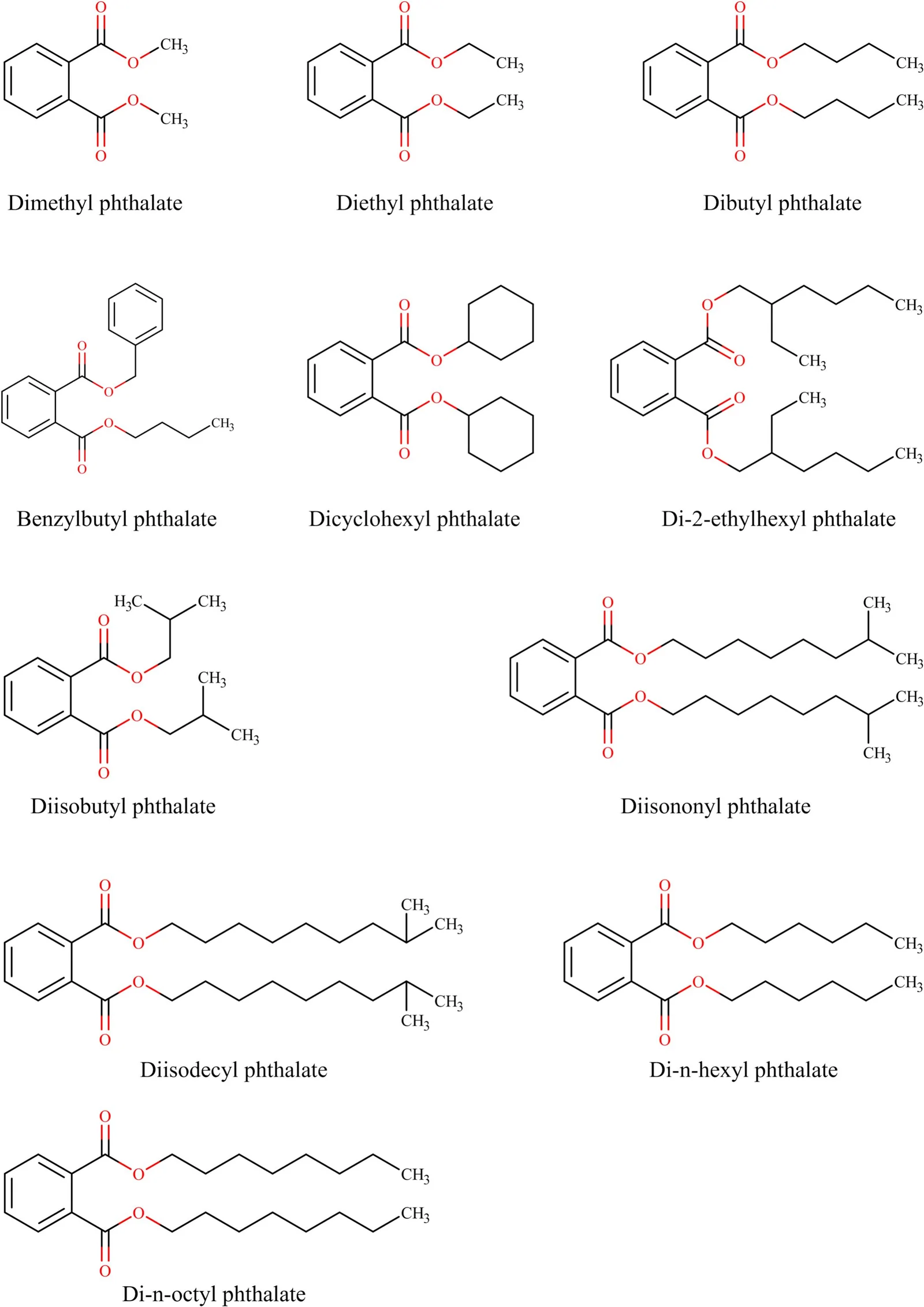
Chemical structures of commonly studied phthalates associated with gastrointestinal toxicity.

## 3 Literature selection methodology and bibliometric analysis

### 3.1 Literature search and eligibility criteria

A systematic literature search was conducted across the PubMed, ScienceDirect, and Scopus databases. Articles published between 2020 and 2026 were retrieved using predefined keywords and Boolean combinations including “Phthalates” OR specific phthalate compounds (“DEP”, “DBP”, “DiBP”, “DEHP”, “DiNP”) combined with “gut microbial composition”, “intestinal barrier integrity”, “immunological barrier”, “enteroendocrine cells hormone secretion”, and “enteric glial cell function”. Seminal studies published prior to this time frame were selectively included when they provided foundational or mechanistically essential evidence relevant to the scope of this review. Records were initially screened based on titles and abstracts, followed by full-text assessment of potentially eligible studies. Inclusion criteria were defined as: (i) investigation of phthalate-induced gastrointestinal toxicity, (ii) use of experimental models (*in vitro* and *in vivo*), and (iii) provision of mechanistic insights into gastrointestinal, microbial, immunological, or neuroendocrine pathways. Studies that did not meet these criteria were excluded.

### 3.2 Bibliometric and keyword co-occurrence analysis

Bibliographic records related to phthalate-induced GI toxicity were retrieved from the Scopus database for the period 2020–2026. A total of 3819 author keywords were extracted and analyzed using VOS viewer (V 1.6.20). Keyword co-occurrence analysis was conducted with a minimum occurrence threshold of 5, yielding 221 keywords that met the inclusion criteria. The bibliometric network visualization generated from this dataset is presented in Fig. 2. Network construction was based on co-occurrence links, with node size proportional to keyword frequency and line thickness representing link strength. Clustering was performed using the VOS viewer association-strength normalization method, and keywords were grouped into clusters based on co-occurrence patterns and network proximity. To maintain conceptual focus and mechanistic relevance, keywords directly related to GI integrity, microbial modulation, immune signaling, neuroendocrine regulation, and enteric cellular function were manually curated, thereby minimizing thematic noise and enhancing interpretability of the resulting clusters.

**Fig. 2:**
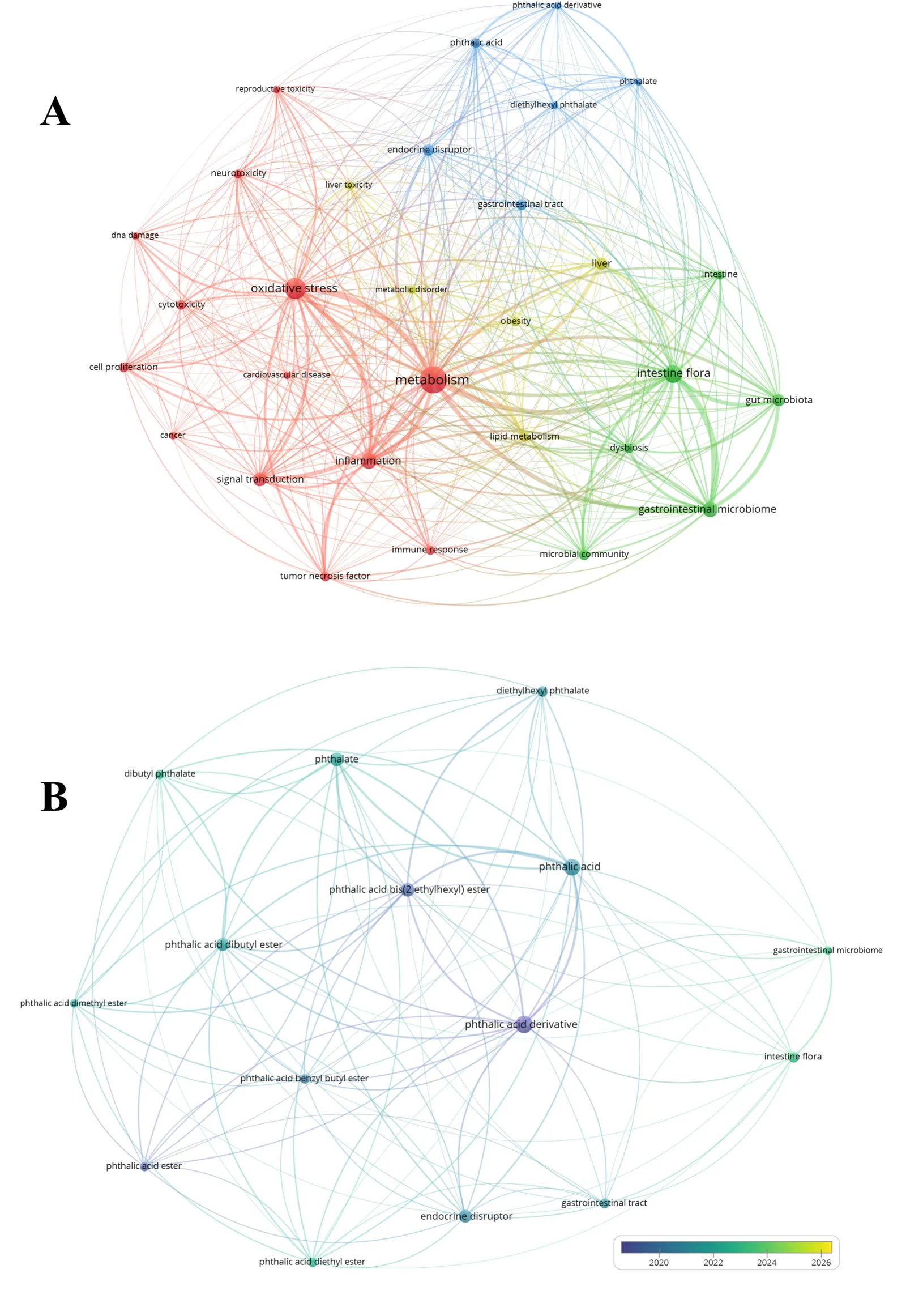
Bibliometric analysis of phthalate-induced gastrointestinal (GI) toxicity research. (A) Keyword co-occurrence network generated using VOSviewer, illustrating major research themes and their interactions in studies related to phthalate-induced GI toxicity. (B) Temporal overlay map showing the evolution of research keywords from 2020 to 2026, highlighting the shifts in research focus.

Fig. 2A illustrates the keyword co-occurrence network associated with phthalate-induced GI tract toxicity. Central nodes, including phthalates, phthalic acid derivatives, and DEHP, exhibit dense interconnections with terms such as gastrointestinal tract, intestinal flora, and gut microbiota, indicating a sustained research focus on gut-specific effects. Closely associated terms, including metabolism, oxidative stress, and inflammation, predominantly cluster within the gastrointestinal context, reflecting their roles as key mechanistic pathways underlying intestinal injury and dysfunction. The strong network connectivity highlights how chemically induced gut alterations act as primary drivers of phthalate-related gastrointestinal toxicity. Fig. 2B presents a temporal overlay of keyword evolution, highlighting shifts in phthalate research. Earlier studies primarily focused on chemical characterization and endocrine-disrupting effects, whereas more recent publications increasingly emphasize gut microbiota, intestinal dysbiosis, and metabolic disorders. This transition reflects a growing recognition of the role of microbiome-mediated mechanisms in phthalate-induced GI toxicity.

## 4 Mechanisms associated with phthalate-impaired gastrointestinal tract function

The GI tract regulates essential physiological processes, including nutrient absorption, digestion, metabolism, pathogen control, and waste disposal (Almamoun et al., 2023). The intestinal barrier plays a crucial protective role by preventing colonization and translocation of toxins and pathogenic microorganisms (Yang et al., 2022a; Cheng et al., 2024). Acute or chronic exposure to phthalates disrupts these functions by altering gut microbiota composition and damaging the intestinal epithelium, ultimately leading to persistent GI dysfunctions and, in severe cases, systemic toxicity (Goyal and Saravanan, 2023). The mechanism underlying phthalate-induced GI impairment, particularly disruption of the physical, chemical, microbial, and immunological barriers, is illustrated in Fig. 3**,** which contrasts healthy and phthalate-impaired GI barriers. Experimental evidence from cellular models is summarized in Table 1, while Table 2 compiles findings from animal studies investigating phthalate-induced GI toxicity. The following sections describe the key dysfunctions caused by phthalate toxicity in the gastrointestinal tract.

**Fig. 3:**
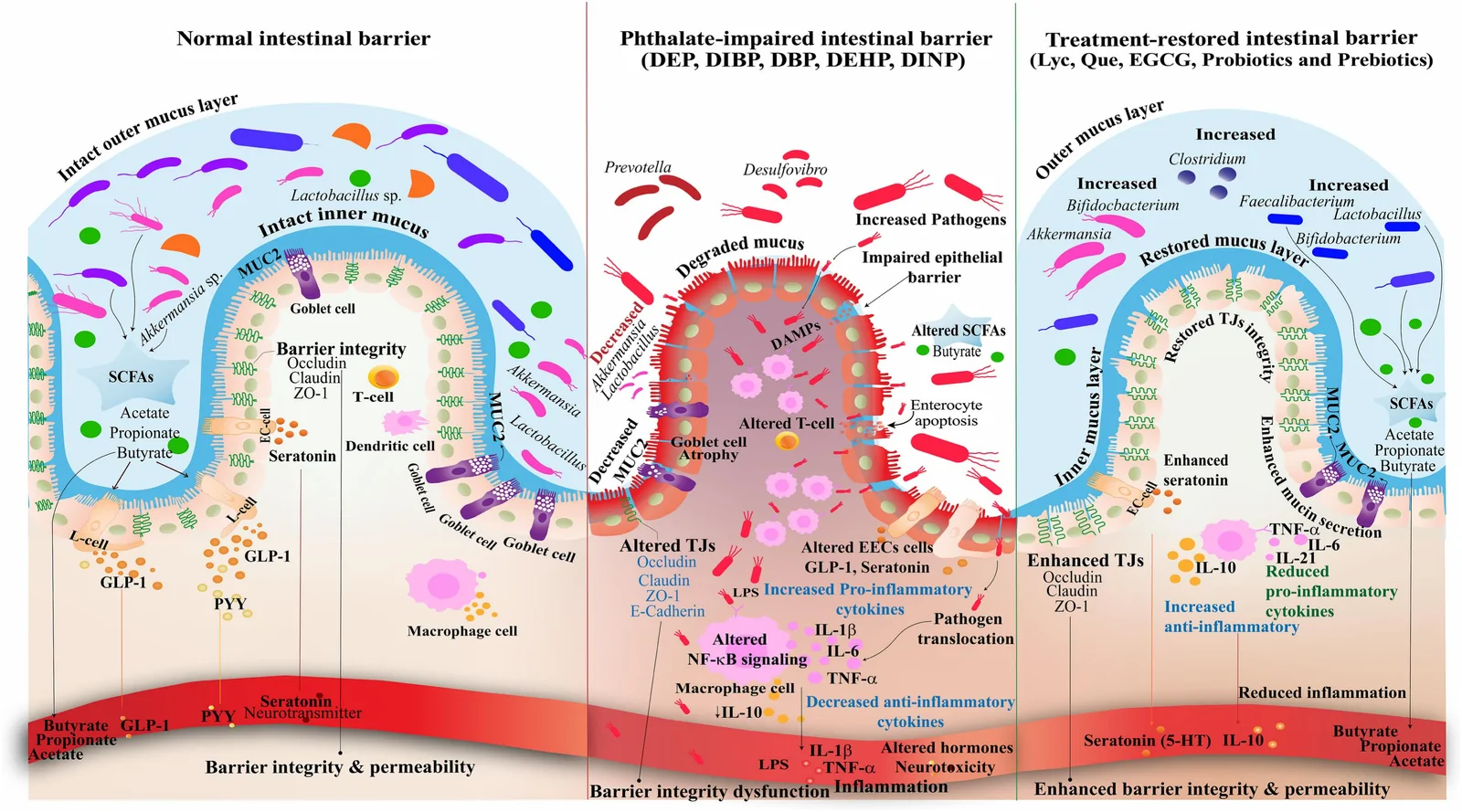
Schematic illustration depicting the intestinal barrier under three conditions: a healthy intestinal barrier with intact epithelial integrity, phthalate-induced impairment of intestinal barrier integrity, and treatment-mediated restoration of intestinal barrier structure and function. Lyc: Lycopene; Que: Quercetin; EGCG: Epigallocatechin gallate; GLP-1: Glucagon-like peptide 1; PYY: Peptide YY; ZO-1: Zonula occludens-1; LPS: Lipopolysaccharide; DAMPs: Damage-associated molecular patterns; IL-1β: Interleukin-1 beta; IL-6: Interleukin-6; IL-10: Interleukin-10; TNF-α: Tumor Necrosis Factor alpha; NF-κB: Nuclear factor kappa-light-chain-enhancer of activated B cells.

**Table 1:** *In vitro* studies on phthalate-induced gastrointestinal toxicity.

Phthalate	Cell line model & exposure dose	Duration of exposure	Endpoints	Key findings	Reference
DiBP+BPA	Caco-2/HT29-MTX cells co-cultured; DiBP (400 mM) and BPA (60 mM)	24 h	o Cell viability o Monolayer integrity o Chemical barrier	↓ Cell viability ↓ Monolayer integrity and permeability ↓ Muc2 protein expression	(Duan et al., 2025)
DBP	Caco-2 (5, 10, 50, 100, and 200 μg/mL	24 h	o Cell viability o Inflammation	↓ Cell viability ↑ *NF-kB*, *FOS*, *Gadd45*, Casp3, and p21-related genes	(Zhao et al., 2021)
DiNP	HT29 cells (0.168, 1.68, and 16.8 μg/mL in fermenter luminal extract)	24 h	o Physical barrier	↓ *Claudin-2, Claudin-4, Occludin, ZO-1, and E-cadherin* mRNA levels ↓ E-cadherin, claudin-4, and claudin-2 protein expression	(Shum et al., 2023)
DEHP	HT29 cells (1.05, 10.5, and 105 μg/mL in fermenter luminal extract)	24 h	o Chemical barrier	↓ *Claudin-2* mRNA levels and protein expression	
DEHP	Colonic organoid cultivation from healthy adult C57BL/6 mice (0.1, 1, 10, and 100 μM)	24 h	o Cell viability o GLP-1, PYY, CCK, and GIP secretion	↓ Cell viability ↓ CCK levels; ↑ GLP-1, GIP, and PYY levels in organoid cell supernatant	(Yue et al., 2024)
DEHP	HT29 cells (100 μM)	24 h	o Serotonin (5-HT) expression and secretion	↓ Serotonin (5-HT) expression and secretion levels	(Wang et al., 2023)
DEHP	IPEC-J2 cells (125 μM)	24 h	o Cell viability o Inflammatory response o Apoptosis	↓ Cell viability ↑ Pro-inflammatory cytokines (IL-1β, IL-6, MCP-1, and TNF-α) mRNA level ↑ Cell apoptosis	(Sun et al., 2024)

**Note:** ↑ denotes an increase, enhancement, or upregulation; ↓ denotes a decrease, reduction, or downregulation.

**Table 2:** *In vivo* studies on phthalates-induced gastrointestinal toxicity.

Phthalate	Rodent model & exposure dose	Duration of exposure	Endpoints	Key findings	Reference
DEP	Gestational and lactational exposure of female Wistar rats (800 mg/kg/d)	38 days, from gestational day 8 to postnatal day 30	o Offspring duodenum histology o Cell proliferation	↑ duodenum villus atrophy ↑ Ki67 protein expression in the duodenum crypt	(Setti Ahmed et al., 2018)
DBP	Female and male ICR mice (50 and 500 mg/kg/d)	10 days	o Duodenum and jejunum histology o Chemical barrier	↑ Villus height in the duodenum and ↓ in the jejunum ↓ Goblet cell number	(Yu et al., 2019)
DBP	Male C57BL/6 J mice (0.1 and 1 mg/kg/d)	10 weeks	o Colon histology o Chemical barrier o Physical barrier o Immune barrier o Microbiota composition	↑ Inflammatory cell infiltration and epithelial degeneration, ↓ Goblet cell number ↓ Claudin1 and occludin protein expression and mRNA levels, ↑ Serum LPS ↓ α-diversity, ↑ Firmicutes/Bacteroidetes (F/B) ratio, ↑ *Prevotella, Desulfovibrio, Sutterella,* and *Bacteroides*; ↓ *Oscillospira, Parabacteroides, Akkermansia, Odoribacter,* and *Helicobacter* relative abundance	(Xiong et al., 2020)
DBP	Male Kunming (KM) mice (500 mg/kg/d)	28 days	o Ileum histology o Chemical barrier o Physical barrier	↓ Villus height and Crypt depth ↓ Goblet cell number and Muc2 protein expression, ↓ E-cadherin protein expression	(Duan et al., 2025)
DBP	Male C57BL/6 N mice (10 and 100 mg/kg/d)	5 weeks	o Microbiota composition o Immune barrier	↓ α-diversity, ↓ *Parabacteroides, Lachnoclostridium, Lachnospiraceae UCG-006, A2, Ruminclostridium 6, Ruminclostridium 9, and Clostridium sensu stricto 1* relative abundance ↓ Leucocyte and T-cell number	(Almamoun et al., 2023)
DiBP	HFD-induced male C57BL/6 J mice (300, 500, and 1000 mg/kg/d)	10 weeks	o Microbiota composition	↑ *Faecalibaculum rodentium* abundance	(Liu et al., 2024)
DEHP	Female C57BL/6 J mice (0.02, 0.2, and 30 mg/kg/d)	34 days	o Colon & Ileum tissue histology o Chemical barrier o Physical barrier o Immune barrier o Cell proliferation	↑ Enterocyte sloughing, inflammatory cell infiltration, and submucosa edema in the colon ↓ *Muc3a* mRNA and in colon, ↓ *Zo-1*, *Occludin* mRNA in colon, ↓ CD3 protein expression in colon, ↑ CD3 protein expression in ileum ↑ TNFα protein expression in colon & ileum ↓ *Ki67* mRNA in colon, ↓ Ki67 protein expression in colon	(Bashir et al., 2022)
DEHP	Male C57BL/6 J mice (50 and 100 mg/kg/d)	20 days	o Immune barrier	↓ Lymphocyte, neutrophil, and monocyte cell numbers in peripheral blood ↓ T-cell number	(Linghu et al., 2024)
DEHP	ICR mice (200 mg/kg/d)	28 days	o Duodenum histology o Chemical barrier o Physical barrier o Immune barrier	↑ Crypt depth and inflammatory cell infiltration; ↓ Villus length and integrity ↓ Goblet cell number; ↓ MUC1 and MUC2 protein expression ↓ Claudin 1, Claudin 5, E-cadherin, N-cadherin, Occludin, ZO-1, and Vimentin protein expression ↑ Proinflammatory cytokines (IL-1β, TNF-α, and IL-6) and ↓ Anti-inflammatory cytokines (IL-10); ↑ NF_Κ_B signaling pathway	(Zhao et al., 2024b)
DEHP	Male C57BL/6 J mice (5 mg/kg/d)	3 weeks	o Intestinal histology o Physical barrier o Immune barrier o Enteroendocrine o Neurotoxicity o Microbiota composition	↑ Crypt swelling and inflammatory cell infiltration ↑ Proinflammatory cytokines (IL-6, TNF-α, and IL-1β) ↓ Serotonin expression and secretion ↓ Learning and memory, ↑ Hippocampus inflammation ↑ *Dubosiella, Bifidobacterium,* and *Lachnospiraceae* relative abundance	(Wang et al., 2023)
DEHP	Female Wistar rats (500 mg/kg/d)	–	o Intestinal histology o Microbiota composition	↑ Inflammatory cell infiltration ↓ Genus *Odoribacter* relative abundance ↓ Butyrate levels	(Yang et al., 2025c)
DEHP	Male C57BL/6 mice (200 mg/kg/d)	35 days	o Microbiota composition o Chemical & Physical barrier o Neurotoxicity	↑ *Bifidobacterium* and *Faecalibaculum*, and ↓ *Akkermansia, Enterorhabdus*, and *Odoribacter* relative abundance ↓ Colon length and goblet cells number; ↓ ZO-1 and Occludin protein expression and mRNA levels ↑ Depression-like behavior and hippocampal neuronal damage	(Chen et al., 2025
DiNP	Male CD-1 mice (0.02, 0.2, 2, 20, and 200 mg/kg)	10 days	o Colon histology o Physical barrier o Immune barrier	↑ Submucosal edema and inflammatory cell infiltration ↓ *Zo-3* mRNA levels ↑ *TNF-α* mRNA levels	(Chiu et al., 2020)
DiNP	Male Wistar rat (200 mg/kg/d)	10 days	o Colon histology o Physical barrier o Immune barrier	↑ Submucosa edema and inflammatory cell infiltration ↓ *ZO-3* mRNA levels ↑ *IL4, IL6, IL13, IL17A, TNF,* and *IFN*γ mRNA levels	(Ahmed et al., 2021)
DiNP	Female CD-1 mice (0.02, 0.2, 2, 20, and 200 mg/kg/d)	10 days	o Chemical barrier o Cell proliferation	↓ MUC2 protein expression ↑ Ki67 protein expression and ↑ *Lyz1* mRNA levels	(Chiu et al., 2021)

**Note:** ↑ denotes an increase, enhancement, or upregulation; ↓ denotes a decrease, reduction, or downregulation.

### 4.1 Interplay among phthalate-induced gut microbiota dysbiosis, barrier dysfunction, and host pathophysiology

Microbial barrier function represents a primary defensive mechanism of the epithelial and mucosal layers, preventing colonization and infection caused by harmful pathogens through the activity of the gut microbiota (Cheng et al., 2024). This function operates alongside chemical, physical, and immunological barriers and is mediated through mechanisms such as resource competition and the production of antimicrobial substances, including short-chain fatty acids (SCFAs), antimicrobial peptides (AMPs), *etc.*, thereby regulating host immune responses (Zhang et al., 2022a). The gut microbiota is often referred to as the “second brain” and plays a crucial role in maintaining host health, neural functions, and nutritional balance (Almamoun et al., 2023).

Microbial barrier function is impaired following phthalate exposure, leading to increased oxidative stress and alterations in microbiota composition, ultimately resulting in pathogen overgrowth and reduced SCFAs synthesis (Yang et al., 2022b; Zhang et al., 2022b; Liu et al., 2024). Firmicutes and Bacteroidetes are the dominant phyla within the gut microbiota and are critically involved in host energy homeostasis, including SCFAs biosynthesis and triglyceride accumulation (Goyal and Saravanan, 2023). Deviations in the Firmicutes/Bacteroidetes (F/B) ratio are widely recognized as a hallmark of gut dysbiosis. Notably, exposure to DEHP has been shown to decrease the F/B ratio, accompanied by enrichment of potentially pathogenic microbial populations and disruption of normal gut metabolic functions (Shum et al., 2023). On the other hand, exposure to DBP has been shown to increase the F/B ratio, leading to impairment of microbial homeostasis and colon inflammation (Xiong et al., 2020). These findings suggest that deviations in the F/B ratio are indicative of gut dysbiosis, highlighting that disruption of microbial equilibrium, rather than the direction of change *per se,* is critical to gut health. Butyrate is a key SCFA that provides energy to colonocytes and plays an essential role in maintaining intestinal hemostasis and immune regulation (Hodgkinson et al., 2023). Disruption of butyrate metabolism, therefore, significantly compromises the intestinal function and barrier. For instance, mice exposed to DBP exhibited increased abundance of pathogenic genera, including *Desulfovibrio*, *Prevotella, Sutterella, Bacteroides,* and *Enterorhabdus*, along with the reduced levels of beneficial genera such as *Parabacteroides, Rumniniclostridium* 6, *Oscillospira, Akkermansia, Odoribacter*, *Helicobacter,* and *Lachnoclostridium* genera (Xiong et al., 2020; Almamoun et al., 2023). Notably, *Desulfovibrio* is a sulfate-reducing bacterium that produces H_2_S in the presence of hydrogen. Excessive sulfide production induces mucosal dysfunction by inhibiting butyrate oxidation in colonocytes, thereby promoting intestinal inflammation (Almamoun et al., 2023; Hodgkinson et al., 2023). Similarly, subchronic exposure to DEHP disrupted lysozyme homeostasis in the small intestine, resulting in elevated levels of pathogenic bacteria and reduced abundance of beneficial symbiotic microbes (Yang et al., 2022b). Furthermore, DEHP exposure induced microbial barrier dysfunction and immune dysregulation in food allergy-induced mice by exacerbating allergic responses (Fan et al., 2025). In addition to gut microbiota dysbiosis, inflammation, and oxidative stress, DEHP causes multiple organ failure at higher doses (Cheng et al., 2024).

Conversely, excessive abundance of certain beneficial microbes may also contribute to host dysfunction. For example, *Faecalibaculum rodentium,* a potent probiotic bacterium in the rodent gut microbiome, exhibits antitumor activity and supports intestinal integrity by producing butyrate and acetate (Yang et al., 2025b). However, DiBP-induced enrichment of *F. rodentium* in obese mice has been linked to the development of cardiovascular disease (Liu et al., 2024). Moreover, impaired gut microbiota may facilitate increased accumulation of xenobiotics in the intestine, leading to increased intestinal permeability. Phthalate exposure has also been shown to disrupt gut microbiota in non-mammalian species. For instance, DiNP exposure significantly altered the gut microbial dynamics in *Eisenia fetida* by suppressing the abundance of the phylum *Chloroflexi* (Zhang et al., 2022b). Additionally, DBP exposure induced sex-specific gut-brain axis dysfunction in zebra fish. Under similar exposure conditions, male zebrafish developed neurological impairments, whereas female zebrafish exhibited severe intestinal dysfunctions. In males, increased abundance of tryptophan-metabolizing bacteria reduced tryptophan availability for serotonin synthesis, impairing gut function and limiting serotonin transport to the brain (Tao et al., 2025). In contrast, female zebrafish displayed elevated expression of *tph2* and serotonin transporter (SERT), suggesting enhanced serotonin transport to presynaptic terminals (Molteni et al., 2010).

Overall, these findings indicate that gut microbiota dysfunction induced by phthalate exposure not only compromises intestinal barrier function but also profoundly affects host neural and immune homeostasis.

### 4.2 Phthalate-impaired intestinal chemical barrier

The mucosal lining of the intestine functions as a chemical barrier that traps toxins and prevents invasion by pathogenic microorganisms (Yang and Yu, 2021). Chronic exposure to toxicants disrupts the mucosal layer through gut dysbiosis and induces excessive production of inflammatory cytokines, leading to tissue inflammation and damage (Zhao et al., 2022). SCFAs, predominantly produced by the gut microbiota, play a crucial role in maintaining the integrity of the chemical barrier. Reduced or inhibited SCFAs production impairs the chemical barrier by decreasing mucin secretion, reducing antimicrobial peptides (AMPs), and disrupting intestinal pH homeostasis (Ma et al., 2022; Mendis et al., 2025). Notably, impaired acetate metabolism has been shown to compromise intestinal integrity and mucin production (Mendis et al., 2025).

Goblet cells are specialized epithelial cells present in both large and small intestines that synthesize mucin, a high molecular weight glycoprotein, forming the mucosal layer that protects the intestinal epithelium (Yang and Yu, 2021). A reduction in goblet cell numbers leads to decreased mucin production, resulting in a thinner mucus layer and increased vulnerability of the intestinal barrier to environmental toxins (Xiong et al., 2020; Bashir et al., 2022; Duan et al., 2025). Among mucins, Muc2 is a secreted, gel-forming mucin produced by goblet cells that constitute the thick mucosal layer; its expression is markedly reduced when goblet cells are damaged. In contrast, Muc2 and Muc3 are transmembrane tethered mucins that form the first line of defense at the epithelial cell surface (Grondin et al., 2020).

Several studies have demonstrated that phthalate exposure disrupts mucosal barrier integrity by altering mucin expression and goblet cell function. For instance, DiBP exposure impaired paneth and goblet cell function, as evidenced by reduced lysozyme and Muc2 expression (Duan et al., 2025). Similarly, DBP exposure significantly reduced goblet cell numbers in the duodenal and jejunal regions of mice, thereby impairing mucus production and barrier function (Yu et al., 2019). In an ovalbumin-induced food allergy mouse model, DEHP exposure reduced goblet cell abundance, suppressed mucin production, and elevated systemic inflammatory markers (Fan et al., 2025).

Moreover, DEHP exposure significantly downregulated Muc1 expression in the mouse ileum and Muc3a expression in the colon, while Muc2 and Muc4 expression remained unchanged (Bashir et al., 2022). These findings suggest that DEHP selectively impairs the glycocalyx layer without directly affecting goblet cell function and further indicate that the colon may be more susceptible to DEHP-induced toxicity than the ileum. In contrast, another study reported that DEHP exposure downregulated both Muc1 and Muc2 expressions in the duodenum, indicating concurrent impairment of transmembrane mucins and goblet cell-derived mucins (Zhao et al., 2024b). Collectively, these observations suggest that the severity of phthalate-induced mucosal toxicity is dependent on exposure concentration, duration, and tissue specificity. Mechanistically, a recent study demonstrated that DEHP exposure enhances cytochrome P450 (CYP450) enzyme activity *via* activation of xenobiotic-sensing receptors and CYP isoforms, thereby inducing Nrf2 signaling pathway in the jejunum in a dose-dependent manner (Yang et al., 2022a). Additionally, diamine oxidase (DAO), a mucosa-derived enzyme and established marker of intestinal integrity, was found to be elevated in zebrafish following DEHP exposure, further confirming mucosal barrier damage (Lyu et al., 2025).

Overall, phthalate exposure disrupts the integrity of the intestinal chemical barrier by impairing SCFA metabolism, mucin production, and goblet cell function in a dose-, duration-, and tissue-specific manner. These alterations exacerbate inflammatory responses and weaken mucosal defenses, thereby increasing intestinal susceptibility to environmental toxins.

### 4.3 Phthalate altered intestinal physical barrier

The intestinal epithelial layer is a transmembrane structure composed of various proteins and receptors that regulate selective transport across the membrane, thereby functioning as a physical barrier. This barrier separates the gut microbiota from the underlying vasculature and prevents antigen-induced inflammation by limiting immune cell activation (Shum et al., 2023). However, this barrier is highly vulnerable to toxins, particularly phthalate exposure, which compromises epithelial integrity by disrupting tight junctions (TJs) and adherens junctions (AJs), and by downregulating key junctional proteins such as occludin, claudin-1, and E-cadherin (Yang et al., 2022b).

Tight junction proteins form a selective seal between adjacent epithelial cells and regulate paracellular transport; disruption of their expression or function increases epithelial permeability and activates immune responses. For example, DBP exposure significantly reduced both mRNA and protein expression levels of *occludin* and *claudin-1* in mice (Xiong et al., 2020). Similarly, DEHP exposure in HT-29 cells decreased *claudin-2* mRNA and protein expression in a dose-dependent manner. In contrast, DiNP exposure resulted in a broader downregulation of both TJ and AJ-associated mRNA, including *CLDN2, CLDN4, OCLN, JAMA, CDH1,* and *TJP1,* even at lower concentrations than DEHP, indicating compound-specific differences in toxicity (Shum et al., 2023).

In addition, DEHP exposure has been shown to inhibit the expression of aquaporin-1 (AQP1), a crucial water-transport protein that maintains intestinal integrity. Suppression of AQP1 contributes to inflammation and barrier dysfunction through activation of the TLR4/MyD88/NF-κB signaling pathway (Zhao et al., 2024b). Beyond molecular alterations, phthalate exposure also induces morphological and pathological damage to intestinal epithelium. For instance, DBP-exposed male Kunming mice exhibited reduced villus height and crypt depth, along with decreased expression of heat shock proteins HSP70 and HSP90, further confirming compromised tight junction integrity and cell damage (Duan et al., 2025). Conversely, DEHP-exposed mice showed reduced villus height and increased crypt depth in the duodenum, resulting in a decreased villus-to-crypt (V/C) ratio in a dose-dependent manner (Yang et al., 2022b). As discussed previously, alterations in villus and crypt morphology impair mucus production and disrupt the gut microbial niche by reducing attachment sites and ecological stability. These morphological changes ultimately compromise microbial function, reduce beneficial microbiota populations, promote pathogen overgrowth, and contribute to immune dysregulation. Furthermore, disruption of TJ and AJ functions leads to epithelial cell inflammation and apoptosis. For instance, exposure to DBP and DEHP in Caco-2 and IPEC-J2 cells, respectively, has been shown to reduce epithelial cell viability and induce inflammation by upregulating pro-inflammatory cytokines mRNA levels, including *IL-1β*, *IL-6*, *MCP-1*, and *TNF-α* as well as to trigger apoptosis through the activation of *NF-kB-*related genes (Zhao et al., 2021; Sun et al., 2024). In line with these findings, exposure to DBP and DEHP in animal models has been reported to induce epithelial cell sloughing and degeneration, along with submucosa edema, accompanied by upregulation of pro-inflammatory cytokines and enhanced epithelial cell apoptosis (Xiong et al., 2020; Bashir et al., 2022; Wang et al., 2023; Zhao et al., 2024b). By contrast, DEP exposure has been shown to mediate duodenal villus atrophy by regulating epithelial cell proliferation (Setti Ahmed et al., 2018), suggesting a compensatory response to toxic injury and epithelial barrier damage.

Overall, phthalate exposure compromises the intestinal physical barrier by disrupting the integrity of TJs and AJs, altering epithelial transport proteins, inducing structural damage to the villus-crypt architecture, and elevating epithelial cell inflammation and apoptosis. These changes increase intestinal permeability, promote immune activation, and exacerbate gut dysbiosis, thereby impairing overall intestinal homeostasis.

### 4.4 Phthalate altered the intestinal immune barrier and host physiology

Exposure to phthalates alters the gut microbiome and significantly impairs both physical and chemical barriers, resulting in the translocation of pathogen and their derivatives, such as lipopolysaccharides (LPS), into the bloodstream. This activates the TLR4 receptors and triggers inflammatory responses through the TLR4/NF-kB and NF-κB/NLRP3/IL-1β/MCL-1 signaling pathways (Xiong et al., 2020; Yang et al., 2022b; Zeng et al., 2024). Consequently, pro-inflammatory cytokine production increases, while the expression of TJs proteins is reduced (Mendis et al., 2025). Furthermore, TJs and AJs play important roles in mediating immune responses and maintaining the intestinal immune barrier. In mice, DBP exposure elevated pro-inflammatory cytokines, including IL-6 and IL-β levels (Xiong et al., 2020). Similarly, DEHP increased serum inflammatory factors, including IgE, IgG, IgG-1, IgG-2, histamine, and MCP-1 (Fan et al., 2025). In addition, DEHP activated the aryl hydrocarbon receptor (AhR), a key mediator involved in sensing exogenous environmental pollutants, along with the NF-κB signaling pathway, leading to intestinal immune cell activation and multiple organ dysfunction (Cheng et al., 2024).

Indeed, phthalate exposure has been reported to induce an imbalance in intestinal immune cells, thereby compromising the intestinal immune barrier. Exposure to DEHP, DBP, and DEP in RAW264.7 cells has been shown to activate macrophages, accompanied by increased expression of inflammatory mediators (COX-2 and iNOS) and elevated levels of pro-inflammatory cytokines, including TNF-α, IL-1β, and IL-6 (Kim et al., 2025). Similarly, DiNP exposure has been observed to activate RAW264.7 macrophages by upregulating pro-inflammatory cytokines while downregulating anti-inflammatory cytokines (Shum et al., 2023). In addition, DBP exposure has been shown to reduce Leucocyte and T-cell counts, as well as total white blood cell numbers, including monocytes and helper T (CD4^+^) cells, in peripheral blood (Almamoun et al., 2023), implying phthalate-induced immune dysfunction, although these effects are not limited to the intestinal immune system. Likewise, DEHP exposure reduces key white blood cell counts, including lymphocytes, monocytes, eosinophils, basophils, and neutrophils, through systemic immune impairment (Linghu et al., 2024).

Collectively, these findings suggest that phthalates compromise intestinal barrier integrity and disrupt immune cell balance, ultimately leading to irreversible systemic damage.

### 4.5 Enteroendocrine dysfunction induced by phthalate exposure

Beyond serving as a barrier against pathogenic microbes, the GI tract plays a crucial role in nutrient digestion and absorption. In addition to nutrient uptake, the hormonal responses mediated by enteroendocrine cells (EECs) are essential for maintaining GI homeostasis and regulating systemic metabolism (Nwako and McCauley, 2024). EECs are a specialized and heterogeneous population of epithelial cells distributed throughout the intestinal lining that sense luminal nutrients and secrete a wide range of hormones. These include enterochromaffin-secreting serotonin, K-cells secreting glucose-dependent insulinotropic polypeptide (GIP), L-cells secreting glucagon-like peptide 1 (GLP-1 and GLP-2), peptide YY (PYY), insulin-like peptide 5 (INSL5), and I-cells secreting cholecystokinin (CCK) (Grundeken and El Aidy, 2025).

Phthalates are endocrine-disrupting chemicals (EDCs), exogenous compounds that can impair endocrine function by binding to hormone receptors, mimicking hormones, or interfering with hormone synthesis, secretion, and regulation. Exposure to phthalates such as DEHP and DiNP has been shown to alter sex-hormone production, including testosterone (Earl Gray Jr., 2023; Yang et al., 2025a).

Although evidence linking phthalate exposure to sustained EEC dysfunction remains limited, microbiota dysbiosis is associated with impaired hormone secretion. The gut microbiota regulates EECs' hormone secretion through a symbiotic relationship, in which the host provides the physiological and metabolic environment (Wozniak et al., 2021). Notably, SCFAs such as butyrate, produced by gut microbiota, have been reported to enhance GLP-1, GIP, and PYY secretion in high-fat diet (HFD)-induced obese mice (Lin et al., 2012). As discussed earlier, phthalate exposure alters the composition and diversity of the gut microbiota, leading to microbial dysbiosis. In female Wistar rats, DEHP exposure induced an imbalance in the gut microbiota, significantly reducing the relative abundance of the genus *Odoribacter* and lowering butyrate levels, potentially impairing gut hormone secretion (Yang et al., 2025c). The genus *Odoribacter* contributes to the production of various metabolites, such as SCFAs, which are closely associated with EEC hormone secretion and GI homeostasis (Li et al., 2025). Similarly, DEHP exposure reduced the relative abundance of *Odoribacter* and *Akkermansia*. *Akkermansia muciniphila*, in particular, has been shown to stimulate GLP-1 secretion in NCI-H716 human L-cells (Arukha et al., 2025). Consistent with these findings, DBP exposure also decreased the abundance of *Odoribacter* and *Akkermansia* in mice (Xiong et al., 2020).

In addition to microbiota-mediated effects, phthalates may also directly affect EEC hormone secretion. Short-term exposure to DEHP significantly increased GLP-1, PYY, and GIP levels in colonic organoid cells, while reducing CCK secretion (Yue et al., 2024). These findings suggest that the effects of DEHP on EEC hormone secretion may be exposure and duration-dependent, although mechanisms underlying these responses remain unclear. In addition, systemic and local intestinal inflammation associated with phthalate-induced epithelial leakage has been linked to altered EEC hormone secretion (Zietek and Rath, 2016). Phthalate exposure, particularly to DEHP, has been associated with mucosal inflammation and elevated levels of pro-inflammatory cytokines, such as TNF-α (Wang et al., 2023; Zhao et al., 2024b). These cytokines can modulate enteroendocrine function by affecting GLP-1 secretion and GLP-2 expression (Gagnon et al., 2015; Tsukahara et al., 2015; Wongkrasant et al., 2020). Together with microbiota-driven SCFA depletion, these indirect inflammation- and microbiota-mediated pathways may contribute to the dysregulation of key metabolic hormones, as illustrated in Fig. 4A. Furthermore, serotonin secretion from enterochromaffin cells was significantly reduced following DEHP exposure in both HT29 cells and mouse models (Wang et al., 2023). This finding suggests that phthalates may disrupt the production of gut-derived neurotransmitters, thereby potentially impairing communication within the gut-brain axis. However, direct toxicological studies using isolated EECs remain limited, and the extent to which phthalates affect EEC function, particularly following chronic exposure, remains unclear, highlighting an important gap for future research.

**Fig. 4:**
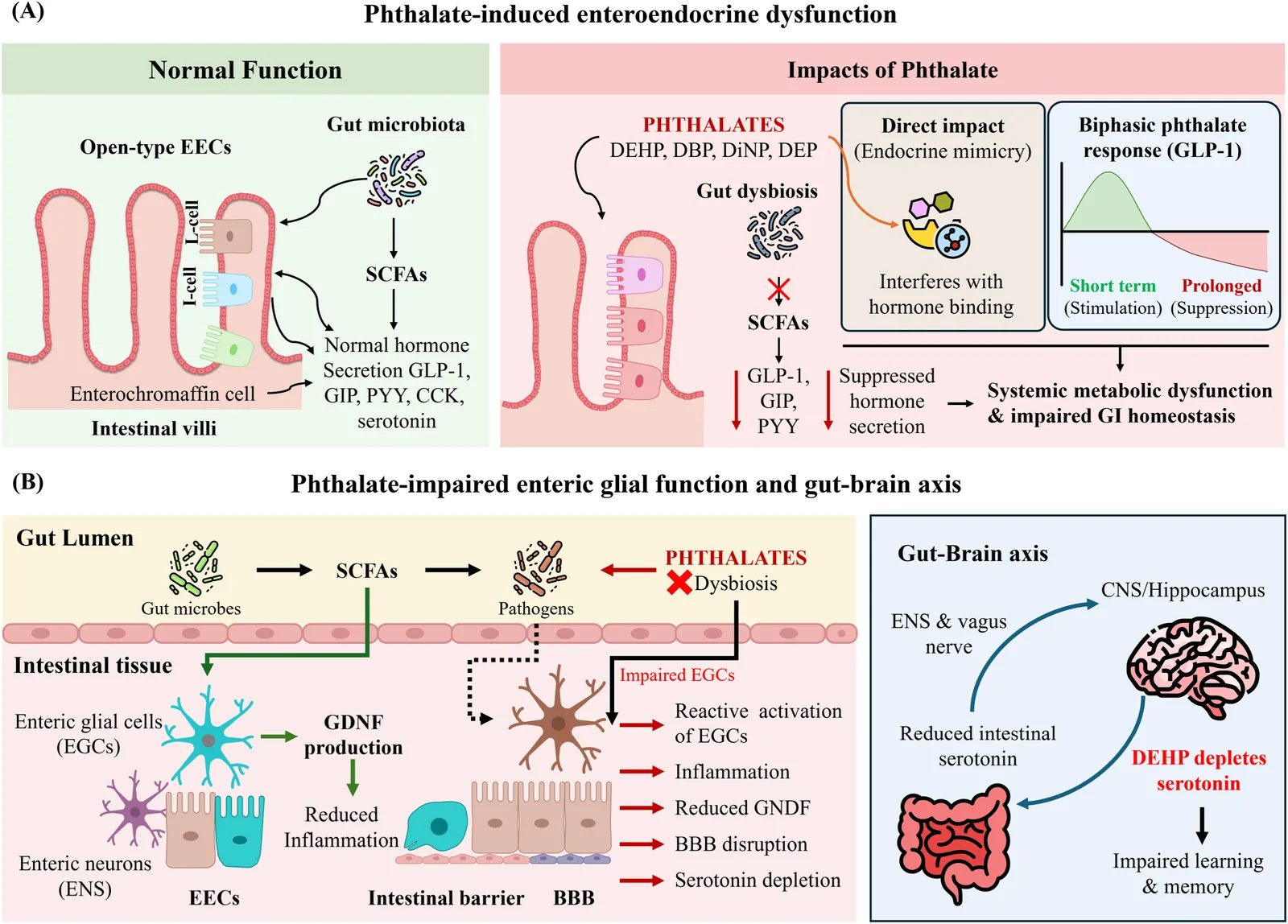
Mechanistic overview of phthalate-induced enteroendocrine and enteric glial dysfunction. (A) Enteroendocrine dysfunction: under physiological conditions, beneficial gut microbiota produce SCFA, which stimulate enteroendocrine cells to secrete metabolic hormones, including GLP-1, GIP, PYY, CCK, and serotonin. Phthalate exposure induces gut dysbiosis and SCFA depletion, suppressing hormone secretion, while endocrine-disrupting activity and the biphasic effects of phthalate, notably DEHP (acute stimulation *versus* chronic suppression of GLP-1), further impair enteroendocrine signaling, contributing to metabolic dysfunction. (B) Enteric glial dysfunction and gut-brain axis disruption: in healthy conditions, microbiota-derived SCFA support EGCs and GDNF production, maintaining intestinal homeostasis. Phthalate-induced dysbiosis promotes EGC activation, intestinal inflammation, reduced GDNF production, BBB disruption, and impaired serotonergic signaling through decreased serotonin synthesis and 5-HT receptor expression, ultimately disrupting vagal communication with the central nervous system and compromising cognitive function.

### 4.6 Phthalate-impaired intestinal enteric glial function

In addition to nutrient absorption, the GI tract is innervated by the enteric nervous system (ENS), which plays a critical role in maintaining gut physiology. Accumulating evidence indicates that extensive plasticity mechanisms triggered by extrinsic cues contribute to ENS heterogeneity. Enteric glial cells (EGCs) are exposed to two distinct microenvironments: internal exposure involving interactions with neurons, immune cells, epithelial cells, and enteroendocrine cells, and external exposure, including nutrients, microbes, and their metabolites.

EECs exist in both open and closed types. Closed-type EECs are positioned near the basal membrane and are indirectly activated through humoral and neural signaling pathways. These cells play a pivotal role in integrating hormonal and neural signals within the body and transmitting them to system physiological responses (Grundeken and El Aidy, 2025). For instance, serotonin (5-HT) secreted by enterochromaffin cells influences learning and memory processes in the central nervous system (CNS). This occurs through the binding of serotonin receptors (5-HT receptors) on the surface of ENS cells, triggering intracellular calcium influx and subsequent cellular activation, ultimately communicating with the CNS *via* the vagus nerve (Barton et al., 2023). Exposure to DEHP has been shown to decrease serotonin synthesis in HT-29 cells, resulting in reduced serotonin levels in mouse serum and the hippocampus. This ultimately results in impaired learning and memory processes (Wang et al., 2023). Consistently, DBP exposure in adult male and female Zebrafish also reduced serotonin synthesis and serotonin receptor expression in the brain tissue, along with decreased serum serotonin levels (Tao et al., 2025).

In addition, EGCs play a crucial role in the gut, functioning similarly to astrocytes in the brain. They contribute to the formation of a gut barrier analogous to the blood-brain barrier (BBB) by supporting neurons and regulating intestinal permeability, epithelial integrity, and immune response (Savidge et al., 2007). Zhao et al. reported that DEHP exposure induces neurotoxicity by enhancing BBB disruption (Zhao et al., 2024a). Furthermore, maternal exposure to monobutyl phthalate (MBP), a primary metabolite of DBP, promotes neural stem cell (NSC) differentiation into astrocytes by regulating *SOX9* and *ATF3* gene expression in mouse offspring, thereby increasing synaptic phagocytosis and cognitive dysfunction (Cui et al., 2025). Similarly, DBP exposure impaired BBB integrity by downregulating ZO-1 and occludin protein expression and activating the NAD^+^/Sirt1/FOXO1a signaling pathway in both mouse models and human brain microvascular endothelial cells (HBMECs) (Liu et al., 2025). However, direct evidence linking phthalate exposure to EGCs-mediated BBB dysfunction remains limited.

Although the bidirectional regulation between enteric glial cells (EGCs) and neuronal function, as well as their relationship with the blood-brain barrier (BBB), is beyond the scope of this review, these interactions have been extensively reviewed elsewhere (Seguella and Gulbransen, 2021). Although direct evidence linking phthalates to specific EGC receptors remains limited, EGCs can undergo reactive activation in the context of mucosal inflammation associated with phthalate exposure, during which they secrete pro- or anti-inflammatory cytokines and chemokines and interact with the gut microbiota to support tissue repair and maintain homeostasis. These interactions are bidirectional and closely linked to immune cells and the microbiota through the secretion of glial cell-derived neurotrophic factor (GDNF) (Mu et al., 2025), which can activate RET tyrosine kinase signaling in group 3 innate lymphoid cells (ILC3) and group 2 innate lymphoid cells (ILC2) (Savidge et al., 2007; Ibiza et al., 2016). Under physiological conditions, baseline levels of these cell populations help maintain mucosal barrier integrity and prevent pathogen entry; however, excessive activation or accumulation may promote tissue inflammation and contribute to IBD pathogenesis (Lee et al., 2019). Consistent with this, a significant increase in EGCs has been reported in the colon following DEHP exposure (Bashir et al., 2022). Furthermore, EGCs can interact with epithelial cells by regulating tight junction proteins, thereby modulating intestinal permeability (Cheadle et al., 2013). Under persistent inflammatory or toxic stress, excessive glial activation may therefore contribute to sustained tissue inflammation and barrier dysfunction. However, the specific molecular crosstalk linking phthalates, EGCs, immune cells, and epithelial cells remains insufficiently characterized and requires further empirical validation. The interconnected cascade linking this phthalate-induced enteric glial activation, BBB disruption, and depleted serotonin signaling to cognitive impairments is illustrated in Fig. 4B. However, direct mechanistic studies are needed to determine whether phthalates directly affect EECs and EGCs and to elucidate the molecular crosstalk among phthalates, microbiota, immune cells, EECs, EGCs, and the intestinal epithelium.

Notably, *Akkermansia muciniphila* has been shown to alleviate cognitive dysfunction by modulating serotonin signaling through the gut-brain axis (Kang et al., 2025). This study further demonstrated that *A. muciniphila* attenuated inflammation by increasing the expression of EGC markers, including Iba-1 and PGP9.5, in colon tissues. In contrast, DEHP exposure reduced the relative abundance of *Akkermansia*, leading to increased colon inflammation and neurotoxicity (Chen et al., 2025).

In summary, the gastrointestinal barrier consists of a cooperative network comprising microbial, chemical, physical, and immunological components that collectively regulate nutrient absorption and prevent the translocation of pathogens and toxicants. Under physiological conditions, the gut microbiota serves as the primary line of defense. It collaborates with downstream barrier components by competing with opportunistic pathogens, maintaining microbial community balance, and producing beneficial metabolites such as short-chain fatty acids (SCFAs) and antimicrobial peptides (AMPs). These microbial metabolites reinforce the chemical barrier, contribute to metabolic and enteroendocrine regulation, regulate host immune responses, and maintain epithelial tight-junction integrity.

Following phthalate exposure, chronic oxidative stress initiates a sequential breakdown of this network. First, phthalates induce microbial dysbiosis, characterized by altered microbial composition and reduced production of SCFA and AMPs, thereby disrupting metabolic homeostasis and enteroendocrine signaling. Second, this chemical barrier failure promotes mucosal dysfunction and pathobiont overgrowth. Third, opportunistic pathogens and unmitigated toxicants compromise the physical barrier by downregulating tight-junction proteins (*e.g.*, occludin and claudin-1), leading to epithelial hyperpermeability (“leaky gut”). Finally, the translocation of luminal antigens and toxicants, such as LPS, across the disrupted epithelium activates mucosal immune cells, promotes immune dysregulation and inflammation, and triggers reactive activation of enteric glial cells. Together, these interconnected disturbances impair enteroendocrine signaling and may contribute to systemic inflammation and toxicity.

## 5 Therapeutic and preventive strategies to mitigate phthalate-induced toxicity

The growing body of evidence discussed above clearly demonstrates the detrimental effects of phthalates on GI toxicity. Despite this, no specific pharmacological agents are currently approved for the treatment of phthalate-induced GI toxicity. Consequently, preventive and therapeutic strategies primarily focus on reducing exposure and on biological mitigation, including lifestyle and dietary modifications, avoidance of phthalate-containing products, and nutritional interventions. Given that dietary phthalate exposure profoundly disrupts gut microbiota composition and intestinal homeostasis, recent research has increasingly emphasized gut-modulating strategies, such as natural bioactive compounds and probiotic supplementation, as promising approaches to attenuate phthalate-induced GI toxicity. In this context, this section summarizes current evidence on therapeutic and preventive interventions and highlights the mechanisms underlying how these strategies alleviate the adverse GI effects of phthalate exposure.

### 5.1 Natural compounds treatment

The integration of natural compounds-based strategies into public health and dietary interventions is increasingly recognized as an effective approach to support gastrointestinal health and reduce the toxic effects of environmental contaminants. Several natural compounds, including lycopene, quercetin, epigallocatechin gallate (EGCG), resveratrol, lutein, *N*-acetylcysteine, melatonin, and folic acid, have been reported to exert protective effects against exposure to environmental toxicants, particularly in the reproductive system (Oyovwi et al., 2025).

Lycopene (Lyc), a carotenoid abundantly present in red and pink fruits and vegetables, has demonstrated significant protective effects against phthalate exposure. Co-administration of Lyc during DEHP exposure significantly mitigated GI toxicity by enhancing SCFA production and restoring gut microbiota homeostasis. This effect was associated with an increased abundance of beneficial bacteria, including *Bifidocbacterium* and *Akkermansia*, leading to improved gut barrier integrity and reduced inflammation (Chen et al., 2025). Additionally, Lyc exhibited anti-inflammatory effects in the colon by suppressing the LPS-TLR4/MyD88 signaling pathway and enhancing EGCs' function, as evidenced by increased HuC/D and Tuj1 expression (Wang et al., 2025).

Quercetin (Que), a flavonoid widely found in fruits and vegetables, has also shown protective effects against DEHP-induced intestinal toxicity. Co-treatment with Que. significantly reduced cytotoxicity and apoptosis in DEHP-induced IPEC-J2 cells, accompanied by suppression of inflammatory cytokines and modulation of antioxidant activity *via* the ROS/ASK1/JNK signaling pathway (Sun et al., 2024). Furthermore, Que. administration improved intestinal barrier function in ulcerative colitis (UC)-induced mice by restoring gut microbiota balance, notably increasing the abundance of *Clostridium* cluster XIVa and *Clostridium* XI (Lei et al., 2025). Similarly, EGCG demonstrated protective effects by enhancing gut microbiota homeostasis and intestinal barrier integrity. Although reports directly linking EGCG to phthalate exposure are limited, EGCG supplementation increased beneficial microbial populations, including *Faecalibaculum* and *Akkermansia*, and improved barrier integrity in polystyrene microplastic-induced mice (Yang et al., 2023). In addition, EGCG co-administration in cyclophosphamide-induced mice significantly reduced intestinal inflammation-related cytokines (TNF-α, IL-10, and IL-21), and upregulated tight junction proteins such as ZO-1, Occludin, and Claudin-1 (Wei et al., 2021).

Collectively, these findings highlight the critical role of natural compounds in mitigating phthalate-induced gastrointestinal toxicity by maintaining gut homeostasis. However, further studies are required to establish their efficacy, optimal dosage, and direct mechanisms of action in the context of phthalate exposure.

### 5.2 Probiotic treatment

In recent years, increasing attention has been directed toward the beneficial effects of probiotics, including *Lactococcus, Clostridium*, and *Lactobacillus* species, as well as lactic acid bacteria (LAB), in mitigating phthalate-induced gut toxicity. Administration of *Clostridium butyricum* in DEHP-induced mice significantly reduced intestinal inflammation-related cytokines (IL-6, TNF-α, and IL-1β), increased serotonin secretion, and restored gut microbiota balance by elevating the abundance of *Clostridium sensu stricto* 1 and *Bifidobacterium pseudolonguma* (Wang et al., 2023). Similarly, *Lactobacillus acidophilus* treatment in DBP-induced Caco-2 cells markedly attenuated inflammatory responses and apoptosis-related gene expression, including *NF-κB, FOS, Gadd45, Casp3,* and *p21* (Sun et al., 2024). *In vivo* study, *Lactobacillus plantarum* TW1–1 alleviated DEHP-induced colon toxicity and gut microbiota dysbiosis by enhanced antioxidant capacity, reducing inflammatory cytokines (TNF-α, IL-1β, and IL-6), and increasing the abundance of beneficial microbial genera, such as *Oscillospira, Paraprevotella, Coprococcus*, *Lactobacillus*, and *Ruminococcus* (Tian et al., 2019).

Moreover, *Lactococcus lactis* subsp. *Lactis* CCFM1018 and *Lactobacillus plantarum* subsp. CCFM1019 was shown to prevent DEHP and MEHP-induced hepatotoxicity and testicular toxicity in mice by modulating gut microbiota homeostasis. This protective effect was associated with increased fecal SCFAs levels, including acetate, propionate, and butyrate, and reduced abundances of harmful bacteria taxa such as *Peptococcaceae, Ruminococcaceae, Bilophila*, *Coprococcus,* and *Lachnospiraceae* (Chen et al., 2022). Additionally, *Lactiplantibacillus plantarum* subsp. P101 alleviated combined DEHP and microplastic-induced gut toxicity in mice by enhancing intestinal barrier integrity through upregulation of Occludin, Claudin-2, and Claudin-3, reducing inflammatory cytokines including TNF-α and IL-1β, and restoring antioxidant activity and microbial balance, particularly by increasing *Akkermansia* abundance (Guo et al., 2025).

Overall, these studies underscore the ameliorative potential of probiotics, particularly LAB, in alleviating phthalate-induced GI toxicity by maintaining gut homeostasis. Nevertheless, further investigations are warranted to determine whether these protective effects are mediated primarily by modulation of the gut microbiota or by direct probiotic-host interactions. These findings collectively highlight probiotics as a promising preventive and therapeutic strategy against phthalate-induced GI toxicity.

### 5.3 Translational feasibility and clinical limitations of mitigation strategies

While natural bioactive compounds (*e.g.*, lycopene, quercetin, and EGCG) and probiotic strains demonstrate protective effects in preclinical rodent models, several translational barriers may limit their direct application to human clinical settings. First, many *in vivo* studies use pharmacological doses of polyphenols and antioxidants to achieve antioxidant, Nrf2-mediated, and anti-inflammatory effects. At sufficiently high doses, some polyphenols may also exhibit adverse or pro-oxidant effects, highlighting the importance of establishing clinically relevant dose–response relationships and long-term safety profiles (Murakami, 2014; Cladis et al., 2022). Second, polyphenols generally exhibit variable, often limited, oral bioavailability due to poor solubility, intestinal and hepatic metabolism, and extensive biotransformation by the gut microbiota (Di Lorenzo et al., 2021). Consequently, the concentrations and chemical forms of native bioactive compounds reaching different intestinal regions may differ substantially from those administered orally, potentially limiting their ability to exert direct local effects in the distal intestine. Third, although probiotic supplementation with *Lactobacillus* and *Bifidobacterium* species can modulate gut microbial composition and function in experimental models, translation to humans is complicated by host- and microbiome-specific colonization resistance. Studies in humans demonstrate that probiotic engraftment and microbiome responses vary considerably among individuals, and that exogenous strains may not readily establish persistent colonization within the gut microbial community (Suez et al., 2018; Zmora et al., 2018). Therefore, probiotic efficacy may depend strongly on the recipient's baseline microbiome, host characteristics, and environmental factors.

To bridge the gap between preclinical efficacy and human application, future research should explore advanced gastrointestinal delivery technologies that improve the stability and site-specific delivery of bioactive compounds and probiotics. Microencapsulation and nanocarrier systems can protect sensitive bioactives and probiotic cells from gastrointestinal stresses and facilitate controlled or targeted release in the intestine (Yao et al., 2020; Razavi et al., 2021). Similarly, pH-responsive delivery systems have shown potential to protect bioactive compounds during upper gastrointestinal transit and to promote their release in the distal intestine (Zeeshan et al., 2019; Kassem et al., 2024). However, the safety, scalability, pharmacokinetics, manufacturing feasibility, and long-term efficacy of such platforms require further evaluation. Ultimately, well-designed, adequately powered randomized controlled clinical trials are necessary to establish the efficacy and safety of these mitigation strategies before they can be considered for clinical or public-health applications.

## 6 Conclusions and future perspectives

To our knowledge, this review is among the few comprehensive studies to systematically examine phthalate-induced gastrointestinal toxicity and the mechanisms underlying intestinal barrier damage. Phthalates primarily enter the gastrointestinal tract through contaminated food and drinking water, where they disrupt intestinal barrier integrity and trigger a range of immunological dysfunctions. This review highlights the mechanistic pathways by which phthalates impair multiple components of the intestinal barrier, including the microbial, chemical, physical, and immunological barriers. In addition, we discuss the roles of endocrine disruption and impaired enteroendocrine and enteric glial function as critical contributors to phthalate-induced gastrointestinal toxicity.

Although several therapeutic strategies, such as antioxidants, prebiotics, probiotics, and natural bioactive compounds, have been investigated for mitigating phthalate-induced GI damage, probiotics and natural compounds appear to be the most effective interventions. These approaches have demonstrated significant protective effects in chronic-exposure models, particularly with long-term administration. However, therapeutic options remain limited for acute or high-dose phthalate exposure, with chemical antioxidants among the few available interventions. As discussed throughout the review, the intestinal barrier functions as an integrated system, and dysfunction of any component can trigger cascading effects that compromise overall gut homeostasis. Among the various barrier systems, the microbial barrier serves as the first line of defense against pathogens and toxicants; therefore, strategies that strengthen gut microbiota composition, such as prebiotic and probiotic supplementation, may offer promising avenues for reinforcing intestinal resilience. Collectively, this review provides a comprehensive overview of phthalate-induced GI toxicity and offers detailed insights into the associated molecular and cellular mechanisms.

From a future perspective, we have highlighted some of the research gaps that require detailed investigations, such as:

• **Mechanistic toxicity:** More comprehensive, systemic, and mechanistic studies on phthalate-induced gastrointestinal toxicity. These should use environmentally relevant doses of phthalates to assess their toxicological effects.
• **Multi-Omics integration:** A multi-faceted research approach, including genomic, epigenomic, transcriptomic, and proteomic analyses, is crucial for a comprehensive understanding of phthalate exposure at the cellular level.
• **Model extrapolation:** Although studies involving cellular and animal models are essential for identifying gastrointestinal toxicity, extrapolating these findings to large-scale epidemiological analyses using organoid models presents notable limitations.
• **Antioxidant Intervention**: Future investigations should also examine the involvement of alternative antioxidant agents responsible for phthalate-impaired gastrointestinal health through comprehensive assessments at Multi-Omics integration.
• **Elucidation of EEC and EGC mechanisms:** Direct *in vitro* and *in vivo* studies are needed to determine whether phthalates directly target enteroendocrine and enteric glial cells and to clarify the molecular crosstalk linking these cells with the microbiota, immune system, and intestinal epithelium.
• **Cumulative and mixture toxicity:** Existing literature focuses predominantly on single-compound exposures; future research should prioritize evaluating the effects of long-term, real-world exposure to complex mixtures of multiple phthalates, including their potential additive or synergistic effects on gastrointestinal barrier integrity and microbial ecology.

Bridging this gap will be essential for the development of innovative, safe, and effective therapeutic strategies to counteract phthalate-induced gastrointestinal toxicity. Furthermore, strict regulatory oversight and improved management practices are urgently needed to limit the misuse of phthalates, particularly within the food processing and pharmaceutical industries. Given that fast foods and packaged foods represent major sources of phthalate exposure, increased regulatory enforcement and public awareness are critical to reducing exposure and mitigating associated health risks.

## Generative artificial intelligence (AI)

During the preparation of this manuscript, the authors used OpenAI's ChatGPT (GPT-4) and Grammarly EDU (V 1.2.254.1880) for language editing to improve readability and clarity. After using these tools, the authors reviewed and edited the content as needed and take full responsibility for the content of the published article.

## CRediT authorship contribution statement

**Choirul Anwar:** Writing – original draft, Conceptualization, Data curation. **Pitchurajan Krishna Perumal:** Writing – original draft, Visualization, Software. **Ping-Chi Hsu:** Supervision, Validation, Methodology. **Ching-Shu Lai:** Resources, Supervision, Validation, Writing – review & editing, Funding acquisition.

## Funding

This study was funded by the National Science and Technology Council (NSTC), Taiwan, R.O.C. (Grant No. 110-2320-B-992-002-MY3; 113-2320-B-992-001).

## Declaration of competing interest

The authors declare the following financial interests/personal relationships which may be considered as potential competing interests: Ching-Shu Lai reports financial support was provided by National Science and Technology Council (NSTC), Taiwan. If there are other authors, they declare that they have no known competing financial interests or personal relationships that could have appeared to influence the work reported in this paper.

## Data Availability

The data are available from the corresponding author (C.S. Lai) upon reasonable request.
